# Evaluating the Economic Impact of Learn‐to‐Swim Programmes: A Cost–Benefit Analysis of the First Lap Voucher Programme in Australia

**DOI:** 10.1002/hpja.70162

**Published:** 2026-02-12

**Authors:** Siyuan Wang, Rona Macniven, Amy Peden, Blake Angell

**Affiliations:** ^1^ The George Institute for Global Health UNSW Sydney Sydney New South Wales Australia; ^2^ School of Population Health, Faculty of Medicine and Health UNSW Sydney Sydney New South Wales Australia

**Keywords:** cost–benefit analysis, drowning prevention, economic evaluation, learn‐to‐swim programme, water competence

## Abstract

**Issue Addressed:**

Swimming is an essential life skill for Australian children. Access to swimming lessons helps develop competence in the water. However, participation in swimming lessons among preschool‐aged children has declined in recent years, particularly following the pandemic. The New South Wales Government introduced the First Lap programme to encourage preschoolers' access to swimming lessons by providing AUS$100 vouchers during the 2021–2022 and 2022–2023 financial years. The programme aimed to increase participation in early swimming education and raise parental awareness of its importance for water safety. We assessed its economic impact from a policy perspective.

**Methods:**

We surveyed registered First Lap providers and participating families to estimate programme benefits. Costs were sourced from the state government, with outcomes reported using standard cost‐benefit metrics. Subgroup analyses by Socio‐Economic Indexes for Areas quantiles examined variations in programme impact across community groups.

**Results:**

A total of 100 providers and 14 126 parents/carers completed the surveys. Our findings suggest direct benefits of AUS$39.3 million against costs of AUS$28 million, yielding AUS$1.40 per dollar invested. Including indirect benefits increased total returns to AUS$62.3 million, or AUS$2.23 per dollar. Benefits consistently exceeded costs in both financial periods, with returns between AUS$1.38 and AUS$2.11 in 2021–2022 and AUS$1.43 to AUS$2.38 in 2022–2023. Subgroup analysis showed direct returns from AUS$1.40 to AUS$1.43 and AUS$2.22 to AUS$2.25 across socio‐economic groups.

**Conclusions:**

We found strong economic support for the programme, with significant benefits across socio‐economic groups.

**So What?:**

The findings suggest that the First Lap programme, providing swimming vouchers to preschoolers, is a cost‐effective initiative to help families access lessons and promote children's safety and health.

## 1 Introduction

Swimming is an essential life skill that provides a range of health and well‐being benefits [1, 2]. Evidence swimming lowers the risk of premature mortality and promotes the development of life‐saving skills for children [3]. Enhancing water competence through swimming education is widely recognised as an essential component of drowning prevention among children, with research demonstrating that participation in learn‐to‐swim programmes can reduce drowning risks for children aged 1–4 by up to 80% in the United States [4, 5]. Enhancing swimming and lifesaving skills across the population is a key strategy in Australia, as outlined in the Australian Water Safety Strategy 2030, which aims to reduce drowning deaths by 50% by 2030 [6]. Increasing participation in swimming education is expected to substantially lower the risk of child drownings and improve water safety outcomes nationwide, with the annual economic benefit of avoided child drownings estimated at approximately AUS$174 million [7]. However, access to swimming lessons remains challenging for many children in Australia. Inadequate swimming skills and limited water safety knowledge continue to be key risk factors for unintentional drowning, particularly among those facing barriers stemming from socio‐economic factors such lesson costs, residence in rural or remote areas, limited lesson availability, health or disability needs and family circumstances [8, 9].

In Australia, unintentional drowning is a significant cause of injury‐related mortality and morbidity among children [10]. Between 2021 and 2023, 620 individuals drowned in Australian waterways, including 88 children under the age of 14 [11, 12]. In addition, during this period, drowning and submersion injuries resulted in 520 hospitalisations, with 280 involving children under 14 [13]. Although drowning rates for children under 5 have declined by over 67% in Australia over the past 25 years, infants and young children remain most at risk, with rates of 0.9 and 1.8 drownings per 100 000 children [13, 14]. In contrast, rates were lower among older children, with 0.5 per 100 000 for those aged 5–9 and 0.3 per 100 000 for those aged 10–14 [13]. Furthermore, a cross‐sectional analysis of children aged 5–12 revealed that nearly 40% of primary school children in Australia do not meet the national standard for adequate swimming skills [15]. Limited accessibility, lack of awareness, and high costs have been identified as significant barriers to accessing formal swimming lessons, particularly for families from lower socio‐economic backgrounds, those residing in rural and regional areas, and those from culturally and linguistically diverse (CaLD) communities [8, 16]. Furthermore, the COVID‐19 pandemic has exacerbated these challenges. During the pandemic, participation in swimming lessons was constrained by widespread pool closures, missed lessons, persistent shortages of qualified instructors, and extended waitlists [17]. In the post‐pandemic period, participation has been challenged by cost‐of‐living pressures, inflation and ongoing financial barriers [17].

To improve access to early childhood swimming lessons, the NSW Government initiated the First Lap voucher programme in December 2021. Eligible parents or carers of children aged 3–6 years received two AUS$100 vouchers, one in each financial year (2021–2022 and 2022–2023). The programme was designed to enhance participation in learn‐to‐swim programmes among preschool‐aged children (aged 3–6 years) and to raise awareness of parents and carers regarding the essential role of early swimming education [18]. During the two financial years, a total of 350 068 vouchers were created for 296 141 individual children, with 277 488 vouchers being redeemed, representing a redemption rate of 71% [18]. To ensure the program's sustainability and inform future policy decisions, it is important to assess whether the benefits generated by the First Lap program outweigh its implementation costs. To assess the economic evidence and guide future government investment decisions, we conducted a comprehensive cost–benefit analysis (CBA) of the First Lap programme. Specifically, our analysis quantified and compared the total benefits and costs associated with the First Lap programme for the 2021–2022 and 2022–2023 financial years from a policy perspective against the baseline scenario of not implementing the programme. This economic analysis was a component of the broader impact and outcome evaluation of the First Lap programme [19]. In our analysis, we adhered to the methodological framework for conducting CBA as outlined by the NSW government [20].

## 2 Methods

### 2.1 Baseline Sociodemographic Data

Baseline sociodemographic data were collected for each child participant through the NSW Office of Sport registration system, which was completed by the parent or carer via an online government platform. Mandatory data fields included age and gender, while other sociodemographic questions provided opt‐out options. The collected data included age (calculated from date of birth), gender (male/female/prefer not to say), disability status (yes/no/prefer not to say), and primary language spoken at home (options included English, Arabic, Cantonese, Greek, Italian, Mandarin, Vietnamese, or a free‐text field for other languages). In addition, residential postcode was collected (assumed to be the same for the parent/carer and the child), which enabled the derivation of Socio‐Economic Indexes for Areas (SEIFA).

### 2.2 Consumer Benefits

Two surveys were administered to parents and carers in July 2022 and April 2023. For the purpose of this analysis, participating parents and carers were considered consumers. While parents were invited to participate in both financial years, only data from the second survey (April 2023) were used in this analysis, as it was the most recent and most representative of the target group of preschool‐aged children. The surveys included contingent valuation questions designed to measure the value that respondents placed on receiving the lessons covered by the voucher and how it affected their participation in swimming lessons. Contingent valuation is a stated preference methodology frequently employed in the economic literature to value non‐market goods [21]. Specifically, parents and carers were asked whether they would have participated in swim lessons regardless of the voucher, the number of lessons covered by the AUS$100 of the First Lap voucher, the total number of lessons their child enrolled in during the voucher period (e.g., school term) and how much they would have been willing to pay for a term or holiday intensive of swimming lessons without the AUS$100 voucher. For respondents who stated that they would participate in swimming lessons regardless of the First Lap voucher, it was assumed they would receive a benefit of $100 (equal to the value of the voucher). For respondents who indicated they only participated because of the first lap voucher, we estimated their perceived value of the $100 voucher from the three data elements stated above (Appendix A, Q1–3). To estimate consumer benefits for the cohort, two groups were considered. For respondents who affirmed that they would have participated in swimming lessons irrespective of the voucher, a valuation of AUS$100 (i.e., the value of the voucher) was assigned to their participation. Otherwise, for those who would not have enrolled without the voucher, their benefits were estimated based on their willingness to pay (WTP) for the equivalent lessons (the contingent valuation approach). Specifically, for this cohort, the perceived benefit for one respondent was calculated as the perceived benefit of attending one swimming lesson multiplied by how many swimming lessons the First Lap voucher covered the cost of.

We assumed that the WTP estimated from the consumer survey was representative of the entire NSW population and the proportion of respondents who indicated they would have enrolled in swimming lessons regardless of First Lap applied across the state. This approach sought to capture both the tangible and intangible benefits of the voucher programme from the participants' perspective, including enhancements in water safety and confidence, reduced drowning risks, increases in physical activity, expanded social interactions and the recreational value from swimming activities without necessitating the need to estimate each component of the programme's benefits separately.

For the parent/carer survey used in this analysis, a total of 14 126 parents and carers completed the second survey (April 2023), representing 15.5% of the 91 135 parents/carers who had consented to be contacted for the programme evaluation. Completion rates varied by sociodemographic characteristics. Higher proportions of parent/carers of non‐Indigenous children (11.7%) completed the survey compared with those of Aboriginal and Torres Strait Islander children (9.4%). Completion was also higher among parent/carers living in higher socio‐economic areas, with 11.9% in SEIFA 4 compared with 10.0% in quartile 1 (lowest). Slightly higher completion was observed among parent/carers living in regional/remote areas (11.9%) than metropolitan areas (11.4%), while completion was broadly similar across child age groups (10.7%–12.2%) and gender (11.5%–11.6%) and slightly lower among children with a disability (10.6%) compared with those without (11.6%).

### 2.3 Provider Benefits

Between November and December 2022, an online survey was distributed to all registered First Lap programme providers via Survey Manager to estimate additional activity generated by the programme, including changes in the number of swim instructors, employment hours, and income attributable to the programme. A copy of the provider questionnaire is included in Appendix A (Q4). Statewide direct and indirect provider benefits were estimated by extrapolating survey results to 200 providers. This approach represents a conservative estimate, assuming that only 200 out of 518 onboarded providers experienced benefits from voucher redemption. Direct benefits were derived from the reported increases in income and wages for staff at aquatic centres, alongside additional profits for swim school providers. These estimates assumed an hourly wage of AUS$31 for swim instructors and AUS$33 for non‐swim staff, with an annual workload of 34 and 37 weeks, respectively [22]. The estimated total provider benefit was calculated by multiplying the aggregate increase in income for swim and non‐swim instructors by the profit margin and the average rate of economic activity growth observed in the survey. This value was then extrapolated to all providers statewide. To capture the broader economic impact, total benefits, including indirect effects such as increased economic activity in industries supporting aquatic facilities, were estimated by applying the average increase in economic activity from the survey to the national average economic output for swim schools in 2021, assuming that aquatic centres contributed AUS$2.8 billion to the national economy in 2021 [23]. Table 1 summarises the main model assumptions. Both provider and parent/carer survey data were cleaned using STATA and analysed using Microsoft Excel.

**TABLE 1:** Identification of model assumptions.

Statewide economic activity was estimated by extrapolating the average increase observed from a survey of 100 responding providers to 200 providers, out of a total of 574 participating swim schools during the evaluation period. This approach represents a conservative estimate, assuming that only 200 out of 518 onboarded providers experienced benefits from voucher redemption. The average swim teacher received an hourly wage of $31, working 34 weeks per year. The average non‐swim teacher received an hourly wage of $33, working 37 weeks per year. Staff salaries took up 30%–60% of swim schools' income. The aquatic industry in Australia generated a total of $2.8 billion in economic output over 2000 aquatic facilities in 2021. The WTP, as estimated from the parent/carer survey, was representative of the entire NSW population participating in the voucher programme. The proportion of respondents who indicated they would have enrolled in swimming lessons regardless of First Lap applied across the state. Both the benefits and costs associated with the programme were short‐ to medium‐term.

### 2.4 Cost Estimates

Total costs associated with running the First Lap programme for the 2021–2022 and 2022–2023 financial years were extracted from the NSW Government Office of Sport financial records. These included costs associated with voucher redemptions, employee expenses, Service NSW (an online portal for managing NSW government tools and accounts), costs associated with running the programme, and other operating expenses.

### 2.5 Outcomes and Sensitivity Analysis

Outcomes were presented using established CBA metrics, including net benefits (the difference between total benefits and total costs) and benefit–cost ratios (the ratio of total benefits to total costs) [20]. We provided two sets of estimates, denoted as the lower estimate and the upper estimate, corresponding to the direct and total benefits received by providers as described above. Due to the short‐to‐medium duration of our analysis, we did not apply a discount rate to our estimates. All prices were reported in Australian dollars. This study followed the Consolidated Health Economic Evaluation Reporting Standards (CHEERS 2022), with the corresponding checklist provided in Appendix B.

To assess the robustness of our findings, we performed sensitivity and scenario analyses by adjusting key parameters to reflect potential variations. Specifically, we modelled hypothetical scenarios of a 15% increase in voucher redemption rates and a 25% increase in administrative costs to determine how these would affect our findings. In addition, we conducted our CBA under the assumption that the programme's benefits were more widely distributed among swim school providers, increasing the economic gains of the First Lap programme to 300 swim schools. Furthermore, to understand the economic impacts of the programme on various socio‐economic subgroups, we stratified respondents by their reported SEIFA quantiles and repeated the CBA using WTP estimates for each SEIFA quartile across the entire population. Finally, we calculated discount ratios by discounting both programme costs and benefits to their present value, using the 2021–2022 period as the baseline and applying annual discount rates of 3%, 5% and 7% (Appendix C).

## 3 Results

Table 2 displays the demographic characteristics of participants who created a voucher in the 2022–2023 financial year according to area‐level socio‐economic status (SES). From baseline characteristics, we observed that younger children were relatively less likely to be in the lowest socio‐economic quintile, with 16.9% of 3‐year olds in SEIFA 1 compared to 19.3% of 6‐year olds in SEIFA 1. Children living with a disability were relatively more likely to be in the lowest socio‐economic quintile, with 22.7% of children with a disability in SEIFA 1 compared to 17.4% of children without a disability in SEIFA 1. Children who spoke a language other than English at home were relatively more likely to be in the lowest socio‐economic quintile, with 22.5% in SEIFA 1 compared to 16.9% of children who spoke English at home. For children living in regional or remote areas, they were relatively more likely to be in the lowest socio‐economic quintile, with 25.2% in SEIFA 1 compared to 15.8% of children living in metropolitan areas.

**TABLE 2:** Demographic characteristics of participants who created a voucher in the 2022–2023 financial year.

	SEIFA 1 (lowest), *n* (%)	SEIFA 2, *n* (%)	SEIFA 3, *n* (%)	SEIFA 4 (highest), *n* (%)
Age				
3 Years	7682 (16.9)	11 906 (26.2)	10 083 (22.2)	15 792 (34.7)
4 Years	9369 (17.8)	13 820 (26.3)	11 489 (21.9)	17 877 (34.0)
5 Years	6826 (18.0)	10 158 (26.8)	8128 (21.4)	12 819 (33.8)
6 Years	1467 (19.3)	2227 (29.3)	1503 (19.8)	2397 (31.6)
Gender				
Male	12 987 (17.6)	19 632 (26.6)	15 933 (21.6)	25 150 (34.1)
Female	12 298 (17.7)	18 374 (26.5)	15 178 (21.9)	23 590 (34.0)
Living with a disability				
Yes	755 (22.7)	1018 (30.7)	686 (20.7)	861 (25.9)
No	24 039 (17.4)	36 443 (26.4)	30 040 (21.8)	47 343 (34.3)
Language spoken at home				
English	21 014 (16.9)	34 970 (28.1)	26 942 (21.7)	41 370 (33.3)
Other	4333 (22.5)	3153 (16.4)	4264 (22.1)	7525 (39.0)
Location				
Metro	18 102 (15.8)	21 522 (18.7)	27 875 (24.3)	47 360 (41.2)
Regional/remote	7245 (25.2)	16 601 (57.8)	3331 (11.6)	1535 (5.3)

### 3.1 First Lap Programme Voucher Redemption and Costs

Across the 2021–2022 and 2022–2023 financial years, a total of 365 109 vouchers were created, with 266 366 vouchers redeemed, reflecting a redemption rate of 73%. In addition, 581 providers were registered, of which 507 (87.3%) redeemed programme vouchers. Table 3 summarises the estimated total costs associated with the First Lap programme for 2021–2022 and 2022–2023. This amounted to approximately AUS$28 million across the two financial years. The majority of these costs were attributed to voucher redemption (AUS$24.6 million), followed by Service NSW costs (AUS$2.2 million), employee expenses (AUS$0.7 million), and other operating expenses (AUS$0.2 million).

**TABLE 3:** Costs associated with the First Lap programme for the 2021–2022 and 2022–2023 financial years.

	2021–2022 Period (millions)	2022–2023 Period (millions)
Voucher redemption costs	AUS$14.1	AUS$10.5
Employee expenses	AUS$0.3	AUS$0.4
Services NSW costs	AUS$1.0	AUS$1.2
Other operating expenses	AUS$0.1	AUS$0.1
Total	AUS$15.8	AUS$12.2

### 3.2 First Lap Programme Benefits

Out of the 518 registered providers at the time of survey distribution, 100 responded to the provider survey, yielding a response rate of 19.3%. Providers reported an average income growth of 13.6%, alongside an increase of 12 additional lessons and 7.7 more swim instructors per centre as a result of the First Lap programme. Assuming conservatively that these benefits were fully realised across 200 swim schools (approximately 39% of total schools across the state), we estimated that the direct economic benefits for swim schools amounted to approximately AUS$6.4 million. Furthermore, when accounting for indirect economic benefits, the total annual benefits associated with the First Lap programme increased to AUS$17.9 million.

For consumer‐side estimates, a total of 14 126 individuals participated in the second parent/carer survey. Among these participants, 98% reported that they would have enrolled in swimming lessons regardless of the AUS$100 voucher. From the contingent valuation questions, we estimated that respondents valued the swimming lessons covered by the AUS$100 voucher to be worth approximately AUS$108. Assuming that this estimate is representative of all participating families in NSW, the estimated total consumer benefits for the two financial years amounted to approximately AUS$26.5 million.

### 3.3 Cost–Benefit Assessment of the First Lap Programme

Table 4 presents the CBA results for the First Lap programme over the 2021–2022 and 2022–2023 financial years. Under the conservative estimate, the lower bound of net benefits over the 2‐year period amounted to AUS$11.3 million, suggesting that the direct economic benefits of the learn‐to‐swim programme outweighed its associated costs. When incorporating indirect benefits into the economic assessment, the total net benefits increased to approximately AUS$34.3 million, corresponding to a benefit–cost ratio of 2.23. Moreover, the analysis demonstrated a positive economic impact for each period individually, with lower and upper benefit–cost ratio estimates ranging from 1.38 to 2.11 for 2021–2022 and from 1.43 to 2.38 for 2022–2023.

**TABLE 4:** Cost‐benefit analysis of the First Lap programme for the 2021–2022 and 2022–2023 financial years.^a^

	2021–2022 Period		2022–2023 Period		Total	
	Lower estimate	Upper estimate	Lower estimate	Upper estimate	Lower estimate	Upper estimate
Provider benefits^a^	6.4	17.9	6.4	17.9	12.8	35.8
Consumer benefit^a^	15.4	15.4	11.1	11.1	26.5	26.5
Total benefits^a^	21.8	33.3	17.5	29	39.3	62.3
Total costs^a^	15.8	15.8	12.2	12.2	28	28
Net benefits^a^	6.0	17.5	5.3	16.81	11.3	34.3
Benefit–cost ratios	1.38	2.11	1.43	2.38	1.40	2.23

^a^ All figures presented in millions (Australian dollar).

Table 5 summarises the key findings from the sensitivity and subgroup analyses. Our analysis suggested that the total benefits of the First Lap programme consistently exceed the associated costs across all considered scenarios. Specifically, with a 25% increase in total administrative costs, lower and upper bound estimates of benefit–cost ratios for the 2 years corresponded to 1.12 and 1.77, respectively. If voucher redemption rates increased by 15%, lower and upper bound benefit–cost ratio estimates were 1.39 and 2.18. Under the assumption that the programme provider benefits extend to 300 swim schools, corresponding lower and upper bound estimates were 1.63 and 2.86, respectively. In addition, subgroup analysis by SEIFA quartiles revealed that the programme was consistently valued across different socio‐economic and geographical cohorts. Specifically, respondents in the third SEIFA quartile reported the highest consumer benefits, with lower and upper bound benefit–cost ratios of 1.43 and 2.25, respectively. In contrast, the lowest benefit–cost ratios were observed by using the WTP estimates from respondents within the second SEIFA quartile, with lower and upper bound estimates of 1.40 and 2.22, respectively. Finally, the benefit–cost ratio estimates under the assumed discount rates of 3%, 5%, and 7% were comparable to the base case scenario, which applied no discounting. Under a 3% discount rate, the total net benefits of the programme were estimated at AUS$11.2 million (lower estimate) and AUS$33.8 million (upper estimate). At a 5% discount rate, total net benefits were AUS$10.8 million (lower estimate) and AUS$32.7 million (upper estimate). Similarly, applying a 7% discount rate resulted in total net benefits of AUS$10.6 million (lower estimate) and AUS$32.1 million (upper estimate).

**TABLE 5:** Results of sensitivity and subgroup analyses—Estimated benefit–cost ratio for each scenario tested.

Scenarios considered	2021–2022 period		2022–2023 period		Total	
	Lower benefit–cost ratio estimate	Upper benefit–cost ratio estimate	Lower benefit–cost ratio estimate	Upper benefit–cost ratio estimate	Lower benefit–cost ratio estimate	Upper benefit–cost ratio estimate
Increase in administrative costs by 25%	1.10	1.68	1.14	1.90	1.12	1.77
Increase in voucher redemption rates by 15%	1.36	2.07	1.42	2.33	1.39	2.18
Increased spread of provider benefits to 300 providers	1.58	2.67	1.70	3.11	1.63	2.86
Using WTP estimated from SEIFA quartile (1) respondents	1.38	2.11	1.43	2.38	1.40	2.23
Using WTP estimated from SEIFA quartile (2) respondents	1.37	2.10	1.43	2.37	1.40	2.22
Using WTP estimated from SEIFA quartile (3) respondents	1.41	2.13	1.45	2.39	1.43	2.25
Using WTP estimated from SEIFA quartile (4) respondents	1.39	2.12	1.44	2.39	1.41	2.24
Discount ratio (3%)	—	—	—	—	1.40	2.22
Discount ratio (5%)	—	—	—	—	1.40	2.23
Discount ratio (7%)	—	—	—	—	1.40	2.23

## 4 Discussion

In this study, we conducted a comprehensive CBA of the First Lap voucher programme for the 2021–2022 and 2022–2023 financial years. Our analysis indicated that the overall economic benefits of the First Lap programme exceeded its costs, even under conservative assumptions. Over the 2‐year period, the lower and upper bound estimates of the benefit–cost ratio were 1.40 and 2.23, respectively. Notably, the results remained robust to variations in discount rates, given the short time frame of the model. In addition, our analysis revealed that consumer benefits were approximately twice as large as the direct benefits to providers, indicating that the primary beneficiaries of the programme were families rather than swim schools. However, when accounting for the indirect economic benefits to swim schools and related industries, the total provider benefits surpassed consumer benefits, highlighting substantial secondary benefits, particularly for supporting industries. Furthermore, the programme's value was consistent across families of varying SESs, as indicated by their SEIFA quartiles, suggesting that the economic benefits were equitably distributed among different socio‐economic groups. The findings of this study demonstrate the positive returns on investing in learn‐to‐swim programmes for children through both the potential reductions in fatal and non‐fatal drownings, as well as the associated physical health benefits.

Globally, fatal and non‐fatal drowning incidents are estimated to result in total economic losses of approximately $4 trillion, averaging $133 billion annually in countries with high drowning burdens, if current trends persist to 2050 [24]. Investing in basic drowning prevention measures, such as providing fundamental swimming and water safety training to children aged 6 years or older, could prevent an estimated 238 000 fatal and 549 000 non‐fatal drownings [24]. In Australia alone, the economic cost of fatal drowning averages AUS$1.24 billion annually [25]. While swimming lessons alone may not eliminate the risk of childhood drowning, enhancing water competence is widely regarded as an essential component of a broader, multi‐layered drowning prevention strategy, and investing in programmes that reduce the barriers to accessing swimming education is an important aspect of this strategy [4]. Moreover, evidence suggests that promoting swimming is associated with a range of public health benefits, including enhanced physical activity and mental well‐being [26]. These collective benefits highlight the substantial public health value of learn‐to‐swim programmes, supporting their implementation that promotes both safety and well‐being. In addition, future research should investigate the cost–benefits of other prevention strategies to inform policymakers in establishing the business case for investment in evidence‐based drowning prevention strategies in resource‐limited settings. For example, research in the United States suggests that proper use of swimming pool isolation fencing among children under 5 years and consistent use of US Coast Guard‐approved life jackets while boating could prevent an estimated 348 drowning deaths annually and avert approximately $4.5 billion in combined medical and mortality costs [27].

The economic evidence presented in this study can inform advocacy for sustained government investment in the First Lap programme, drawing parallels with other voucher‐based health promotion programmes that have demonstrated successful impact and economic value for children. In NSW, the Active Kids programme provided universal vouchers to support participation in organised sport and physical activity among school‐aged children (aged 4.5–18 years) [28]. Evaluation of the programme has shown that the vouchers increased children's participation in organised sport and physical activity [28]. Economic analyses further suggest that over the funded period (2018–2023) the Active Kids programme generated approximately AUS$0.59 billion in benefits, compared with about AUS$0.57 billion in costs, demonstrating modest but positive economic value [29]. Although the original Active Kids programme concluded in January 2024, it has been continued in a modified form through the Active and Creative Kids initiative, which provides targeted vouchers to support children's participation in physical and creative activities [30]. In the United States, a voucher‐based programme that provided free summer day camp to children from disadvantaged households averted 0.0917 body mass index *z*‐score gain per child at a cost of $1307, corresponding to an incremental cost‐effectiveness ratio of approximately $1463 per 0.1 body mass index *z*‐score averted [31]. Although limited, these findings demonstrate how economic evidence from voucher‐based programmes can strengthen the policy case for government investment in preventive and health‐promoting initiatives such as First Lap.

In our CBA, we evaluated the programme by incorporating short‐ to medium‐term, as well as long‐term, intangible and tangible benefits. While the programme was implemented during the 2021–2022 and 2022–2023 financial years, its impact and potential benefits may extend beyond this timeframe. The contingent valuation method adequately captures these benefits from the consumer perspective, including opportunities for social interactions and leisure, access to water safety knowledge and skills, increased physical activity, enhanced mental well‐being and reduced drowning risks. This methodology provides an upper‐bound estimate of the total benefits associated with the programme by prompting participants to assign a monetary value reflecting their WTP. Alternative economic evaluation methodologies, such as cost‐of‐illness, could be employed to estimate the benefits linked to reduced drowning incidents but may not encompass additional benefits related to health and leisure [32]. In addition, their use in assessing learn‐to‐swim programmes would require more robust epidemiological evidence demonstrating the connection between drowning reduction and participation in swim lessons [5]. While our findings, along with the broader evaluation of the First Lap programme, indicated that the vouchers were more effective in reaching families with prior experience in swimming lessons, evidence from the First Lap programme evaluation showed that approximately 25% of voucher redemptions were for children who had not participated in the previous 12 months. This underscores the opportunity for better resource allocation of the programme to better target those who face barriers to access while also supporting the continued participation of current participating families [18]. From a societal perspective, enhancing access to swimming lessons and addressing barriers, particularly for families with disabilities, those from lower socio‐economic backgrounds, residents of regional and rural areas, and CaLD families, helps to mitigate disparities in access to swimming, accounting for an additional societal benefit of the programme [16, 33].

This study has several strengths and limitations. First, it contributes to the relatively small body of literature on the CBA of drowning prevention interventions. Due to data constraints, we relied on certain strong economic assumptions. Second, the contingent valuation methodology employed did not permit a detailed examination of the components of consumer benefits, particularly the long‐term benefits associated with reductions in drowning incidents. Further research utilising alternative economic evaluation methods, such as cost‐of‐illness estimations, may be required to address this gap. Third, potential reporting bias in the surveys of swim schools and parents/carers may have skewed the results in favour of the programme. Fourth, our findings are based on a single state in a high‐income country, which may limit their generalisability to other Australian states or contexts. Finally, relevant to all economic modelling studies, the accuracy of our model was constrained by its simplifying assumptions and the quality of the inputted data.

## 5 Conclusion

Our CBA of the First Lap voucher programme demonstrated that its economic benefits outweighed its associated costs, with every dollar invested yielding a return from AUS$1.40 to AUS$2.23. These findings remained consistent across various sensitivity analyses. The economic evidence will help to inform governments in making evidence‐based decisions regarding the programme's continuation and potential expansion, ensuring optimal resource allocation to maximise public health benefits and reduce disparities in access to swimming lessons.

## Funding

This study was funded by the New South Wales Government (RG214380).

## Ethics Statement

Ethics approval for the First Lap evaluation and this study was granted by the University of New South Wales Human Research Ethics Advisory Panel (ID: HC220282).

## Conflicts of Interest

The authors declare no conflicts of interest.

## Supporting information


**Appendix A.** Provider and consumer questionnaire.

**Appendix B.** CHEERS 2022 checklist.

**Appendix C.** Present value equation.

## Data Availability

Research data are not shared.
