# Supplementary material for: Evaluating the Economic Impact of Learn‐to‐Swim Programmes: A Cost–Benefit Analysis of the First Lap Voucher Programme in Australia
Source: Health Promot J Austr. 2026 Feb 12;37(2):e70162. doi: 10.1002/hpja.70162 (PMC12895295; doi:10.1002/hpja.70162)
Supplement: Supplementary file 1 — Appendix A. Provider and consumer questionnaire. [file HPJA-37-0-s003.docx]

**Appendix A. Provider and consumer questionnaire**

| Q1. How many swimming lessons did the First Lap voucher ***cover the cost of***? E.g. child does one term of lessons at $200 for 10 lessons ($100 First Lap voucher *covered* 5 of these 10 lessons) OR child does five private lessons at $50 per lesson ($100 First Lap voucher *covered* 2 of these 5 lessons) |
| --- |
| ™ 1-2 lessons |
| ™ 3-4 lessons |
| ™ 5-6 lessons |
| ™ 7-8 lessons |
| ™ 9 or more lessons |

| Q2. How many swimming lessons did your child ***sign up for*** in the time period (e.g. school term) in which you redeemed the First Lap voucher? E.g. child does one term of lessons at $200 for 10 lessons ($100 First Lap voucher covered 5 of these 10 lessons) |
| --- |
| ™ 1-2 lessons |
| ™ 3-4 lessons |
| ™ 5-6 lessons |
| ™ 7-8 lessons |
| ™ 9 or more lessons |

| Q3. How much would you be willing to pay for one term or holiday intensive period of swimming lessons if you ***didn’t have*** a $100 voucher? |
| --- |
| ™ $0 |
| ™ up to $100 |
| ™ up to $150 |
| ™ up to $200 |
| ™ up to $250 |
| ™ over $250 |
| ™ over $300 |

| Q4. Has the First Lap voucher program resulted in any of the following changes in learn to swim lesson operation at your venue? |
| --- |
| a) Increased number of teachers employed |
| b) Increased hours for existing staff (swim teachers) |
| c) Increased hours for existing staff (non-swim teachers) |
| d) Increased swim school income |
