# Supplementary material for: Evaluating the Economic Impact of Learn‐to‐Swim Programmes: A Cost–Benefit Analysis of the First Lap Voucher Programme in Australia
Source: Health Promot J Austr. 2026 Feb 12;37(2):e70162. doi: 10.1002/hpja.70162 (PMC12895295; doi:10.1002/hpja.70162)
Supplement: Supplementary file 2 — Appendix B. CHEERS 2022 checklist. [file HPJA-37-0-s002.docx]

**Appendix B. CHEERS 2022 checklist**

| **Item** | **Guidance for Reporting** | **Reported in section** |
| --- | --- | --- |
| Title | Identify the study as an economic evaluation and specify the interventions being compared. | P1 |
| Abstract | Provide a structured summary that highlights context, key methods, results and alternative analyses. | P2 |
| Background and objectives | Give the context for the study, the study question and its practical relevance for decision making in policy or practice. | P3 |
| Health economic analysis plan | Indicate whether a health economic analysis plan was developed and where available. | P3-4 |
| Study population | Describe characteristics of the study population (such as age range, demographics, socioeconomic, or clinical characteristics). | P4-5 |
| Setting and location | Provide relevant contextual information that may influence findings. | P4-5 |
| Comparators | Describe the interventions or strategies being compared and why chosen. | P5 |
| Perspective | State the perspective(s) adopted by the study and why chosen. | P5 |
| Time horizon | State the time horizon for the study and why appropriate. | P5 |
| Discount rate | Report the discount rate(s) and reason chosen. | P9-10 |
| Selection of outcomes | Describe what outcomes were used as the measure(s) of benefit(s) and harm(s). | P9 |
| Measurement of outcomes | Describe how outcomes used to capture benefit(s) and harm(s) were measured. | P7-10 |
| Valuation of outcomes | Describe the population and methods used to measure and value outcomes. | P7-10 |
| Measurement and valuation of resources and costs | Describe how costs were valued. | P9 |
| Currency, price date, and conversion | Report the dates of the estimated resource quantities and unit costs, plus the currency and year of conversion. | NA |
| Rationale and description of model | If modelling is used, describe in detail and why used. Report if the model is publicly available and where it can be accessed. | P9-10 |
| Analytics and assumptions | Describe any methods for analysing or statistically transforming data, any extrapolation methods, and approaches for validating any model used. | P8-10 and Appendix |
| Characterizing heterogeneity | Describe any methods used for estimating how the results of the study vary for sub-groups. | P9-10 |
| Characterizing distributional effects | Describe how impacts are distributed across different individuals or adjustments made to reflect priority populations. | P9-10 |
| Characterizing uncertainty | Describe methods to characterize any sources of uncertainty in the analysis. | P9-10 |
| Approach to engagement with patients and others affected by the study | Describe any approaches to engage patients or service recipients, the general public, communities, or stakeholders (e.g., clinicians or payers) in the design of the study. | NA |
| Study parameters | Report all analytic inputs (e.g., values, ranges, references) including uncertainty or distributional assumptions. | P8-9 and appendix |
| Summary of main results | Report the mean values for the main categories of costs and outcomes of interest and summarise them in the most appropriate overall measure. | P8-15 |
| Effect of uncertainty | Describe how uncertainty about analytic judgments, inputs, or projections affect findings. Report the effect of choice of discount rate and time horizon, if applicable. | P13-15 |
| Effect of engagement with patients and others affected by the study | Report on any difference patient/service recipient, general public, community, or stakeholder involvement made to the approach or findings of the study | NA |
| Study findings, limitations, generalizability, and current knowledge | Report key findings, limitations, ethical or equity considerations not captured, and how these could impact patients, policy, or practice. | P14-18 |
| Source of funding | Describe how the study was funded and any role of the funder in the identification, design, conduct, and reporting of the analysis | P19 |
| Conflicts of interest | Report authors conflicts of interest according to journal or International Committee of Medical Journal Editors requirements. | P19 |
