# Supplementary material for: Evaluating the Economic Impact of Learn‐to‐Swim Programmes: A Cost–Benefit Analysis of the First Lap Voucher Programme in Australia
Source: Health Promot J Austr. 2026 Feb 12;37(2):e70162. doi: 10.1002/hpja.70162 (PMC12895295; doi:10.1002/hpja.70162)
Supplement: Supplementary file 3 — Appendix C. Present value equation. [file HPJA-37-0-s001.docx]

**Appendix C. Present value equation**

$$Present value= \frac{Future value}{{(1+r)}^{t}}$$

Where r is the annual discount rate and t is the time in years.
